# Transporter Gene Regulation in Sandwich Cultured Human Hepatocytes Through the Activation of Constitutive Androstane Receptor (CAR) or Aryl Hydrocarbon Receptor (AhR)

**DOI:** 10.3389/fphar.2020.620197

**Published:** 2021-01-21

**Authors:** Congrong Niu, Bill Smith, Yurong Lai

**Affiliations:** Drug Metabolism, Gilead Sciences Inc., Foster City, CA, United States

**Keywords:** gene induction, transporters, hepatocytes, constitutive androstane receptor, aryl hydrocarbon receptor

## Abstract

The induction potentials of ligand-activated nuclear receptors on metabolizing enzyme genes are routinely tested for new chemical entities. However, regulations of drug transporter genes by the nuclear receptor ligands are underappreciated, especially in differentiated human hepatocyte cultures. In this study, gene induction by the ligands of constitutive androstane receptor (CAR) and aryl hydrocarbon receptor (AhR) was characterized in sandwich-cultured human hepatocytes (SCHH) from multiple donors. The cells were treated with 2,3,7,8-tetrachlorodibenzo-p-dioxin (TCDD), omeprazole (OP), 6-(4-chlorophenyl)imidazo[2,1-b][1,3]thiazole-5-carbaldehyde O-(3,4-dichlorobenzyl)oxime (CITCO) and phenobarbital (PB) for three days. RNA samples were analyzed by qRT-PCR method. As expected, CITCO, the direct activator, and PB, the indirect activator of CAR, induced CYP3A4 (31 and 40-fold), CYP2B6 (24 and 28-fold) and UGT1A1 (2.9 and 4.2-fold), respectively. Conversely, TCDD and OP, the activators of AhR, induced CYP1A1 (38 and 37-fold), and UGT1A1 (4.3 and 5.0-fold), respectively. In addition, OP but not TCDD induced CY3A4 by about 61-fold. Twenty-four hepatic drug transporter genes were characterized, and of those, SLC51B was induced the most by PB and OP by about 3.3 and 6.5 fold, respectively. Marginal inductions (about 2-fold) of SLC47A1 and SLCO4C1 genes by PB, and ABCG2 gene by TCDD were observed. In contrast, SLC10A1 gene was suppressed about 2-fold by TCDD and CITCO. While clinical relevance of SLC51B gene induction or SLC10A1 gene suppression warrants further investigation, the results verified that the assessment of transporter gene inductions are not required for new drug entities, when a drug does not remarkably induce metabolizing enzyme genes by CAR and AhR activation.

## Introduction

There are about 450 membrane transporters divided into two superfamilies, the solute carrier (SLC) and the ATP Binding Cassette (ABC) transporters. Membrane transporters transport ions, essential nutrients, hormones etc. across a biological membrane. Among these membrane transporters, nearly thirty of them are involved in drug transport and currently are designated as clinically relevant drug transporters (International Transporter et al., 2010; Hillgren et al., 2013). The liver is the most important organ in the body for drug metabolism, accounting for about 70% of drugs and their metabolite elimination in humans (Patel et al., 2016). Many transporters are expressed on the sinusoidal and canalicular membrane of hepatocytes to transport poorly permeable endogenous substances into and out of the liver. About 20 of these membrane proteins are also involved in hepatic uptake and biliary excretion of a wide variety of prescription drugs and their metabolites (Vildhede et al., 2015), and play a key role in determining drug pharmacokinetics (PK) and hepatic exposure. Therefore, fluctuations of expressions and functional activities of drug transporters can result in changes of plasma level and/or liver exposure of substrate drugs. For example, active hepatic uptake mediated by sinusoidal transporters e.g., organic anion transporting polypeptides (OATP) can be the rate-determining step of drug elimination for the substrates that are either metabolically stable or unstable (Hagenbuch and Meier, 2004). The functional inhibition or induction of transporter gene expression can cause drug-drug interactions (DDIs) with co-administration drugs that are substrates of the transporters (International Transporter et al., 2010; Hillgren et al., 2013). Given the critical roles played by hepatic transporters in systemic clearance and liver exposure, it is important to understand the factors affecting transporter expression. Therefore, characterizing transporter gene regulation is a crucial step to improve the understanding of transporter-mediated drug absorption, disposition and elimination, and subsequently their association to drug efficacy and toxicity.

It is well-documented that metabolizing enzymes can be induced by activation of various cytoplasmic and orphan nuclear receptors, such as the pregnane X receptor (PXR) and constitutive androstane receptor (CAR). For example, phase I cytochrome P450 3A (CYP3A), CYP2C and phase II uridine diphosphate glucuronosyltransferase 1A1 (UGT1A1) are induced by PXR ligands (Niu et al., 2019). Similarly, CAR ligands induce CYP2B, 3A, 2C and UGT enzymes (Yang and Wang, 2014). Unfortunately, details in transporter gene regulations by these nuclear receptors remain sparse in the literature (Mottino and Catania, 2008; Tolson and Wang, 2010), and systemic evaluations have not been conducted to the same extent as metabolic enzyme inductions, especially in differentiated hepatocyte cultures with right transporter localizations. Recently we investigated transporter gene induction by rifampin, a known prototypical activator of PXR for enzymes and efflux transporter genes, in sandwich-cultured hepatocytes (Niu et al., 2019). Since sandwich-cultured human hepatocytes (SCHH) consist of collagen Magrigel™ on both sides of the hepatocytes, which allow the differentiation of hepatocytes to form the bile ducts, the hepatocytes can restore the expression of drug metabolizing enzyme and transporter proteins, and most importantly, with correct localization of hepatic drug transporters (Kimoto et al., 2012a; Kimoto et al., 2012b). We also observed similar induction patterns of metabolic enzyme genes by rifampin between sandwich-cultured hepatocytes and plated hepatocytes (Niu et al., 2019). Interestingly, hepatic transporter gene induction by the PXR ligand rifampin was generally not significant in both human and monkey hepatocytes, except for SLC51B, a greater than 10-fold induction was observed (Niu et al., 2019).

In order to further elucidate the regulation of hepatic transporter genes by CAR and AhR activation, dose-response induction studies were conducted in sandwich-cultured human hepatocytes from multiple donors treated with AhR or CAR activators. Omeprazole (OP) and 2,3,7,8-tetrachlorodibenzo-p-dioxin (TCDD) were selected as AhR activators, and 6-(4-chlorophenyl)imidazo[2,1-b][1,3]thiazole-5-carbaldehyde O-(3,4-dichlorobenzyl)oxime (CITCO) and phenobarbital (PB) were used as the direct and indirect activators of CAR, respectively. The gene expressions were quantitated by real-time quantitative reverse transcription polymerase chain reaction (qRT-PCR).

## Materials and Methods

### Ethics Statement

Cryopreserved primary human hepatocytes isolated from deceased donor livers were purchased from *In Vitro* ADMET Laboratories (Columbia, MD 21045) and BioIVT (Westbury, NY). Pre-experiments were conducted to confirm the induction of CYP genes in selected hepatocyte lots. The donor demographics is listed in Table 1. Consent was obtained from the human donor or the donor's legal next of kin for use of these samples and their derivatives for research purposes using IRB-approved authorizations.

**TABLE 1:** Primary human hepatocyte donor demographics.

PHH donor	AOS	OQA	YTW	HH1117	HH1142
Gender	Female	Male	Male	Male	Female
Age	53	62	19	31	27
Race	Caucasian	American indian	Caucasian	Caucasian	Caucasian
Cause of death	Anoxia 2nd to cardiovascular	Head trauma/Blunt injury/Accident, Non-MVA	Head trauma 2nd to Trauma/MVA	Anoxia 2nd to drug intoxication	Head trauma
BMI	24.5	25.8	18.7	27.44	25
Smoking	1ppd x 43 years	Smoked 1 pack of cigarettes per day x 20 years–none in last 6 months	No	No	No
Alcohol	1–2 glasses of wine 4/month for 40 years	Drank 12 beers/day x last 20 years	No	Yes	No
Substance abuse	Heroin one time 18 months ago, cocaine weekly 20 years ago, meth 5 years ago, marijuana daily x 43 years	No	No	Yes	No
Medical history	COPD, cardiomyopathy, HTN for last 18 months	HTN, GERD, anxiety, osteoarthritis, tonsillectomy, appendectomy, vasectomy, broken foot 2 years ago, MRSA 3 years ago–cortisone for shoulder pain	Pituitary gland issue–took growth hormones for 2.5 years, stopped 2 years ago. EBNA IgG+	Allergies (non-specific)	Chest trauma
Infectious diseases	HBV^-^, HCV^-^, HIV^-^, CMV^+^	HBV^-^, HCV^-^, HIV^-^, CMV^+^	HBV^-^, HCV^-^, HIV^-^, CMV^+^	HBV^-^, HCV^-^, HIV^-^, CMV^-^	HBV^-^, HCV^-^, HIV^-^, CMV^+^

### Chemicals and Reagents

Omeprazole (OP), 2,3,7,8-tetrachlorodibenzo-p-dioxin (TCDD), 6-(4-chlorophenyl)imidazo[2,1-b][1,3]thiazole-5-carbaldehyde O-(3,4-dichlorobenzyl)oxime (CITCO) and phenobarbital (PB) were purchased from Sigma-Aldrich (St. Louis, MO). Matrigel™ (phenol red free) was obtained from Corning (Bedford, MA). Collagen I coated 96-well plates were obtained from Life Technologies (Carlsbad, CA). The hepatocyte thawing medium (UCRM™) was purchased from *In Vitro* ADMET (Columbia, MD). The hepatocyte plating medium (*InVitroGRO*™ CP medium), incubation medium (*InVitroGRO*™ HI medium) and *Torpedo* ^*TM*^ Antibiotic Mix were from BioIVT (Westbury, NY). All oligonucleotide primer and probe sets were manufactured by Life Technologies (Table 2).

**TABLE 2:** Information of primer and probe sets manufactured by Life Technologies.

Gene	ID
CYP1A1 TaqMan^®^ gene expression assay	Hs01054796_g1
CYP1A2 TaqMan^®^ gene expression assay	Hs00167927_m1
CYP2B6 TaqMan^®^ gene expression assay	Hs04183483_g1
CYP3A4 TaqMan^®^ gene expression assay	Hs00604506_m1
CYP2C9 TaqMan^®^ gene expression assay	Hs02383631_s1
UGT1A1 TaqMan^®^ gene expression assay	Hs02511055_s1
SLC10A1 TaqMan^®^ gene expression assay	Hs00161820_m1
SLC15A1 TaqMan^®^ gene expression assay	Hs00192639_m1
SLC16A1 TaqMan^®^ gene expression assay	Hs01560299_m1
SLC22A1 TaqMan^®^ gene expression assay	Hs00427552_m1
SLC22A7 TaqMan^®^ gene expression assay	Hs00198527_m1
SLC22A9 TaqMan^®^ gene expression assay	Hs00971064_m1
SLCO1B1 TaqMan^®^ gene expression assay	Hs00272374_m1
SLCO1B3 TaqMan^®^ gene expression assay	Hs00251986_m1
SLCO2B1 TaqMan^®^ gene expression assay	Hs01030343_m1
SLCO4C1 TaqMan^®^ gene expression assay	Hs00698884_m1
ABCB1 TaqMan^®^ gene expression assay	Hs00184500_m1
ABCB11 TaqMan^®^ gene expression assay	Hs00994811_m1
ABCC2 TaqMan^®^ gene expression assay	Hs00960489_m1
ABCC3 TaqMan^®^ gene expression assay	Hs00978452_m1
ABCC4 TaqMan^®^ gene expression assay	Hs00988721_m1
ABCC5 TaqMan^®^ gene expression assay	Hs00981089_m1
ABCC6 TaqMan^®^ gene expression assay	Hs01077866_m1
ABCG2 TaqMan^®^ gene expression assay	Hs01053790_m1
SLC47A1 TaqMan^®^ gene expression assay	Hs00217320_m1
SLC22A5 TaqMan^®^ gene expression assay	Hs00929869_m1
SLC29A1 TaqMan^®^ gene expression assay	Hs01085706_m1
SLC29A2 TaqMan^®^ gene expression assay	Hs02513021_s1
SLC51A TaqMan^®^ gene expression assay	Hs00380895_m1
SLC51B TaqMan^®^ gene expression assay	Hs01057182_m1
Beta actin endogenous control (VIC™/TAMRA™ probe, primer limited)	

### Sandwich-Cultured Human Hepatocytes and Treatment of AhR or CAR Activators

Cryopreserved human hepatocytes were quickly thawed at 37°C and then transferred into hepatocyte thawing medium. Cells were pelleted at 100 × *g* for 10 min and then resuspended in hepatocyte complete plating medium (CP medium). The cells were then seeded at 65,000 cells per well in 96-well collagen I coated plates. After cell attachment (4–6 h post-seeding), CP medium was exchanged for ice-cold complete incubation medium (HI medium) containing BD Matrigel™ at the concentration of 0.25 mg/ml. The cells were then incubated overnight at 37°C in a humidified 5% CO_2_ incubator. The HI medium was replaced by the fresh HI medium containing TCDD (0.08–50 nM), OP (0.08–50 μM), CITCO (0.008–5 μM), PB (1.6–1,000 μM) or dimethyl sulfoxide (DMSO) only. The media containing TCDD, Omeprazole, CITCO, PB or DMSO were subsequently changed daily for a total of three days. Cell death or loss of adhesion of hepatocytes during or after treatment were examined by microscopy. In addition, the β-actin mRNA levels were assessed to confirm that the treatments of transcript activators didn’t show significant cytotoxicity.

### Real-Time Quantitative Reverse Transcription Polymerase Chain Reaction (qRT-PCR)

A RNeasy 96 Kit (Qiagen) was used to extract the total cellular RNA from sandwich cultured hepatocytes in 96-well plates following the manufacturer's instructions. A total volume of 200 μL RNA was obtained via the column elution provided in the RNeasy kit. Five μL RNA extraction was mixed with TaqMan® Fast Virus 1-Step Master Mix (Life Technologies) in a final reaction volume of 20 μL and qRT-PCR was achieved using a QuantStudio 6 Flex Real-Time PCR System (Life Technologies), following the manufacturer's instructions. β-actin mRNA expression in the SCHH was used as a house-keeping gene for the normalization of target gene expressions. The mRNA levels in SCHH treated with AhR or CAR activators were expressed by a fold change, compared to the gene expressions without the treatment. The 2^−ΔΔCt^ method (Livak and Schmittgen, 2001) was used to analyze the relative changes in gene expression, where the Ct is the cycle number at which the fluorescence in the reaction crosses the preset arbitrary threshold; the ΔCt represents the difference between the Ct target and reference and the ΔΔCt is the difference between the ΔCt of the test and the ΔCt of the preassigned control. Gene expression for each condition is expressed as a fold-change of mean ± standard deviation (SD) from three independent donors (each performed in triplicate). All primer and probe sets were purchased from Life Technologies (Table 2). When concentration-dependent induction observed, the *in vitro* induction mRNA data were fitted using the sigmoid 3-parameter equation in GraphPad Prism (La Jolla, CA) to estimate the *in vitro* concentration of inducer that produced half the maximum induction values (EC_50_).

## Results

### Induction of Metabolizing Enzyme Genes by TCDD, OP, CITCO and PB

**FIGURE 1:**
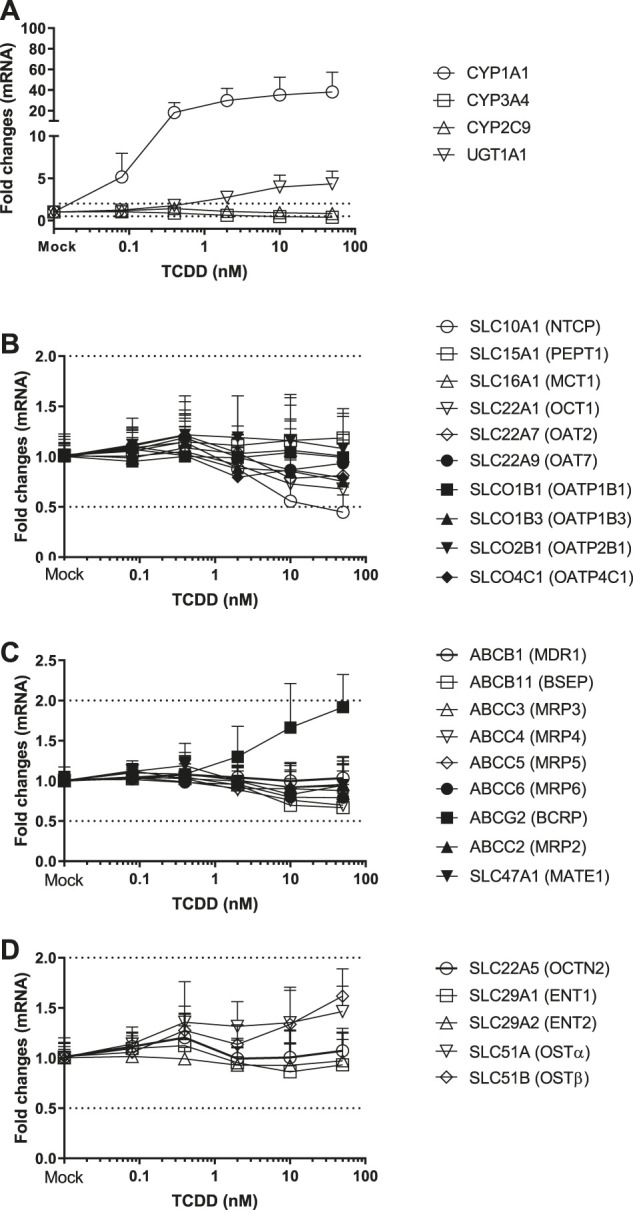
Induction of metabolizing enzyme and transporter mRNA by TCDD. The concentration-dependent induction studies were conducted in SCHH (lot HH1117, HH1142 and YTW) treated with TCDD at the concentrations ranging from 0.08 to 50 nM. The gene expressions were quantitated by qRT-PCR. Gene expression for each condition is expressed as a fold-change of mean ± standard deviation (SD) from three independent donors (each performed in triplicate). Dot lines indicate the change of 2-fold.

The potential induction of metabolizing enzyme genes was investigated in three donors of cryopreserved human hepatocytes. The inductions of metabolizing enzymes including CYP1A1, CYP1A2, CYP3A4, CYP2C9, CYP2B6 and UGT1A1 were used as positive or negative controls for CAR and AhR activators. SCHH were incubated with TCDD (0.08–50 nM), OP (0.08–50 µM), CITCO (0.008–5 µM) and PB (1.6–1,000 µM) for 72 h. TCDD induced CYP1A1 and UGT1A1, to a maximal increases of 38- and 4.3-fold, respectively (Table 3). The TCDD induction was concentration-dependent (Figure 1A), and the EC_50_ was estimated to be 0.5 and 1.56 nM for CYP1A1 and UGT1A1, respectively (Table 4). Likewise, CYP1A1, CYP3A4 and UGT1A1 mRNA inductions by OP were observed in a concentration-dependent manner to a maximum of 37, 61 and 5.0-fold increase at 50 μM, respectively (Table 3). The EC_50s_ of OP induction were 3.34, 7.37, and 28.7 µM for CYP1A1, CYP3A4 and UGT1A1, respectively (Table 4). The mRNA of CYP2B6, CYP3A4 and UGT1A1, but not CYP1A2 was induced by CAR activators, CITCO and PB. The average of 24, 31 and 2.9-fold increase of CYP2B6, CYP3A4 and UGT1A1 mRNA expression were detected in the hepatocytes treated with CITCO at 5 µM (Table 3), with the EC_50_ of 0.16, 1.14 and >5 μM, respectively (Table 4). Similarly, PB induced CYP2B6, CYP3A4 and UGT1A1 mRNA expressions by about 40, 28 and 4.2-fold at the highest concentration tested (1,000 µM) (Table 3), and the EC_50s_ were 726, 343 and 639 μM, respectively (Table 4). Although robust enzyme inductions were detected in SCHH treated with activators of AhR or CAR, it is worth noting that the donor-to-donor variabilities were observed among three lots of human hepatocytes. The CYP3A4 fold induction by CITCO (5 µM) in hepatocytes from the donor OQA was nearly 8-times greater than that of the donor AOS (Supplementary Figure 1). In addition, a greater than 50% reduction of CYP3A4 mRNA (<0.5-fold) by TCDD was detected in only two donors among three lots of hepatocytes, resulting in an average reduction of about 0.4-fold (Table 3) (Supplementary Figure 1).

**TABLE 3:** Changes in gene expression of CYP enzymes and drug transporters in sandwich- cultured human hepatocytes treated with CAR or AhR activators.

	TCDD	OP	CITCO	PB
CYP1A1	38.1 ± 19.2	37.0 ± 17.8	ND	ND
CYP1A2	ND	ND	1.8 ± 0.5	1.3 ± 0.4
CYP3A4	0.4 ± 0.2	61.2 ± 40.5	31.0 ± 22.4	40.5 ± 21.6
CYP2C9	0.8 ± 0.3	2.2 ± 0.6	ND	ND
CYP2B6	ND	ND	24.7 ± 6.6	28.2 ± 20.9
UGT1A1	4.3 ± 1.5	5.0 ± 1.2	2.9 ± 1.6	4.2 ± 1.9
ABCB1 (MDR1)	1.0 ± 0.3	1.3 ± 0.2	1.0 ± 0.3	1.3 ± 0.6
ABCB11 (BSEP)	0.7 ± 0.3	0.7 ± 0.2	0.6 ± 0.2	1.1 ± 0.3
ABCC2 (MRP2)	0.9 ± 0.3	1.2 ± 0.2	1.4 ± 0.6	1.5 ± 0.3
ABCC3 (MRP3)	1.0 ± 0.2	1.0 ± 0.1	1.3 ± 0.4	1.0 ± 0.4
ABCC4 (MRP4)	0.7 ± 0.2	1.4 ± 0.1	1.0 ± 0.3	1.8 ± 0.4
ABCC5 (MRP5)	0.9 ± 0.3	1.0 ± 0.2	1.1 ± 0.5	1.2 ± 0.4
ABCC6 (MRP6)	0.8 ± 0.2	0.7 ± 0.1	1.6 ± 0.6	1.3 ± 0.3
ABCG2 (BCRP)	2.0 ± 0.4	1.7 ± 0.2	1.4 ± 0.3	1.6 ± 0.3
SLC47A1 (MATE1)	0.9 ± 0.3	0.9 ± 0.1	1.0 ± 0.4	2.2 ± 0.7
SLC10A1 (NTCP)	0.5 ± 0.2	0.3 ± 0.1	0.8 ± 0.5	1.4 ± 0.7
SLC15A1 (PEPT1)	1.2 ± 0.3	0.9 ± 0.2	1.6 ± 1.0	1.1 ± 0.5
SLC16A1 (MCT1)	1.0 ± 0.2	1.6 ± 0.3	1.0 ± 0.2	1.1 ± 0.2
SLC22A1 (OCT1)	0.7 ± 0.3	0.7 ± 0.2	0.8 ± 0.3	1.5 ± 0.6
SLC22A7 (OAT2)	0.8 ± 0.2	0.6 ± 0.2	1.1 ± 0.7	0.5 ± 0.2
SLC22A9 (OAT7)	0.9 ± 0.2	0.6 ± 0.1	0.8 ± 0.4	0.6 ± 0.2
SLCO1B1 (OATP1B1)	1.0 ± 0.4	1.4 ± 0.4	1.5 ± 0.3	1.5 ± 0.8
SLCO1B3 (OATP1B3)	0.8 ± 0.5	0.6 ± 0.2	0.7 ± 0.4	0.9 ± 0.2
SLCO2B1 (OATP2B1)	1.1 ± 0.4	1.6 ± 0.2	1.3 ± 0.5	1.8 ± 0.9
SLCO4C1 (OATP4C1)	0.8 ± 0.3	0.8 ± 0.2	1.1 ± 0.5	2.3 ± 0.6
SLC22A5 (OCTN2)	1.1 ± 0.2	1.2 ± 0.4	1.0 ± 0.3	0.8 ± 0.3
SLC29A1 (ENT1)	0.9 ± 0.4	1.0 ± 0.3	1.3 ± 0.6	0.9 ± 0.2
SLC29A2 (ENT2)	1.0 ± 0.2	1.4 ± 0.3	1.0 ± 0.3	0.9 ± 0.3
SLC51A (OSTα)	1.5 ± 0.3	1.0 ± 0.2	0.9 ± 0.5	0.9 ± 0.2
SLC51B (OSTβ)	1.6 ± 0.3	6.5 ± 1.4	1.2 ± 0.3	3.4 ± 1.4

**TABLE 4:** The EC_50_ of gene induction.

Genes	TCDD (nM) (95% CI)	OP (µM) (95% CI)	CITCO (µM) (95% CI)	PB (µM) (95% CI)
CYP1A2	ND	ND	NI	NI
CYP1A1	0.50 ± 0.11 (0.27∼0.73)	3.34 ± 1.39 (0.55∼6.1)	ND	ND
CYP2B6	ND	ND	0.16 ± 0.04 (0.14∼0.31)	726 ± 655 (313∼1,157)
CYP3A4	NI	7.37 ± 7.59 (0.1∼22)	1.14 ± 0.53 (0.63∼1.6)	343 ± 198 (216∼543)
CYP2C9	NI	NI	ND	ND
UGT1A1	1.56 ± 0.40 (0.75∼2.4)	28.7 ± 7.32 (14∼43)	>5 (ND)	639 ± 444 (488∼1,157)

NI, not induction; ND, not determined.

### Induction of Transporter Genes by TCDD, OP, CITCO and PB

**FIGURE 2:**
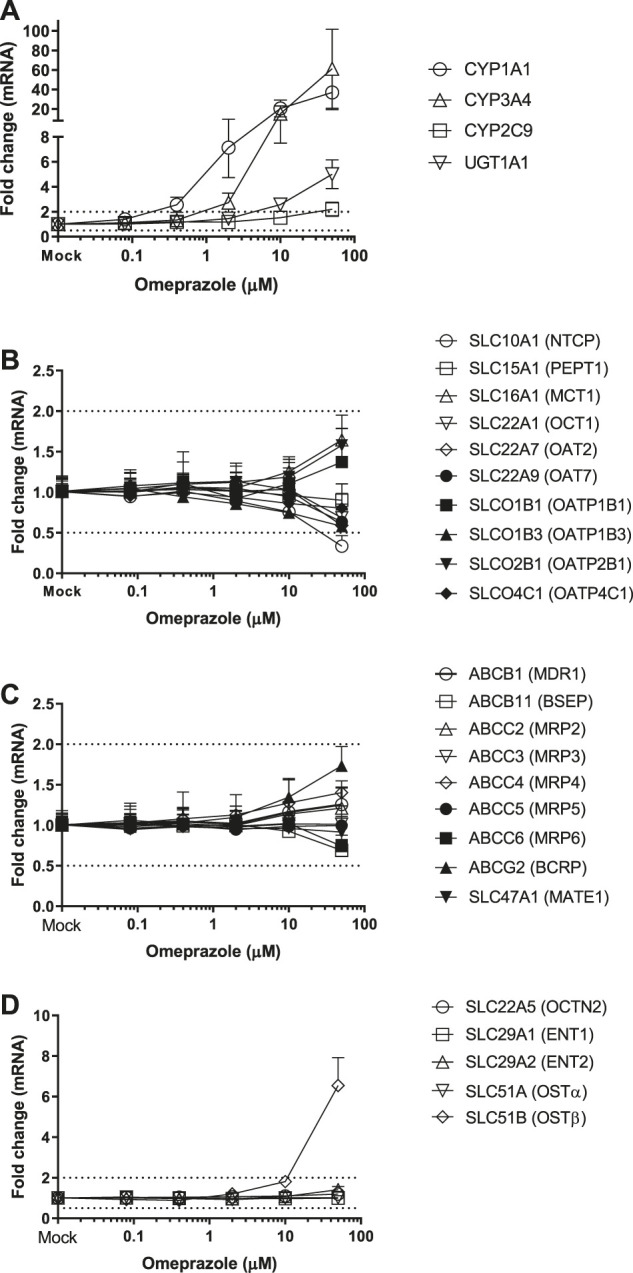
Induction of metabolizing enzyme and transporter mRNA by OP. The concentration-dependent induction studies were conducted in SCHH (Lot HH1117, HH1142 and YTW) treated with OP at the concentrations ranging from 0.08 to 50 μM. The gene expressions were quantitated by qRT-PCR. Gene expression for each condition is expressed as a fold-change of mean ± standard deviation (SD) from three independent donors (each performed in triplicate). Dot lines indicate the change of 2-fold.

**FIGURE 3:**
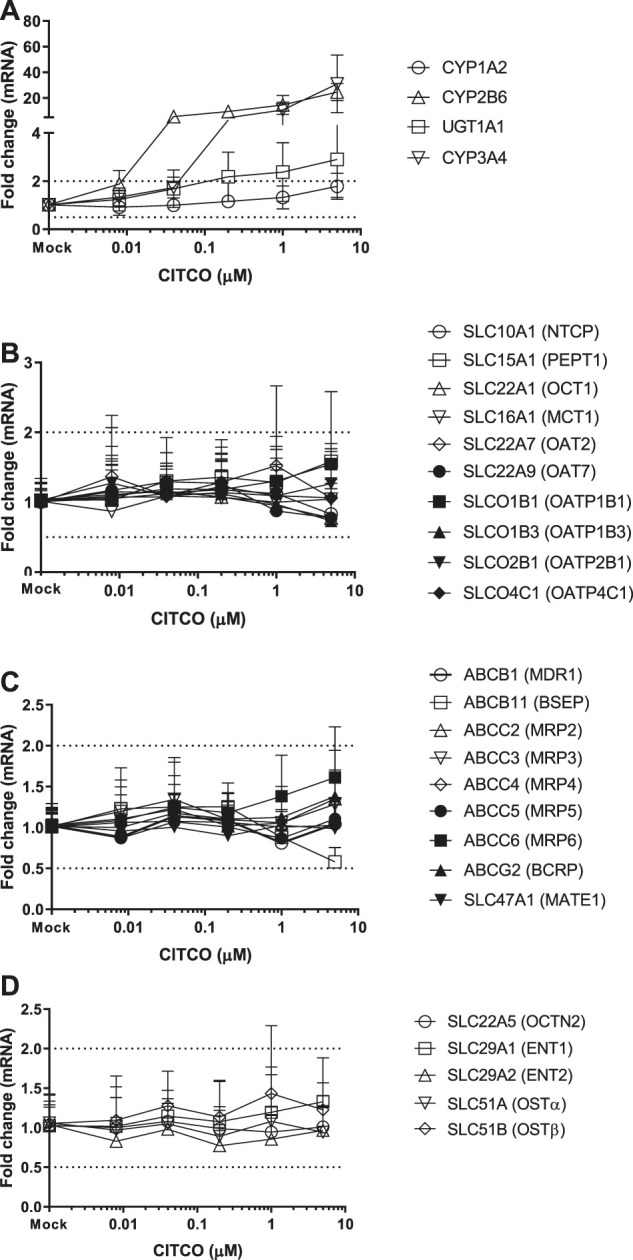
Induction of metabolizing enzyme and transporter mRNA by CITCO. The concentration-dependent induction studies were conducted in SCHH (Lot AOS, OQA and YTW) treated with CITCO at the concentrations ranging from 0.008 to 5 μM. The gene expressions were quantitated by qRT-PCR. Gene expression for each condition is expressed as a fold-change of mean ± standard deviation (SD) from three independent donors (each performed in triplicate). Dot lines indicate the change of 2-fold.

**FIGURE 4:**
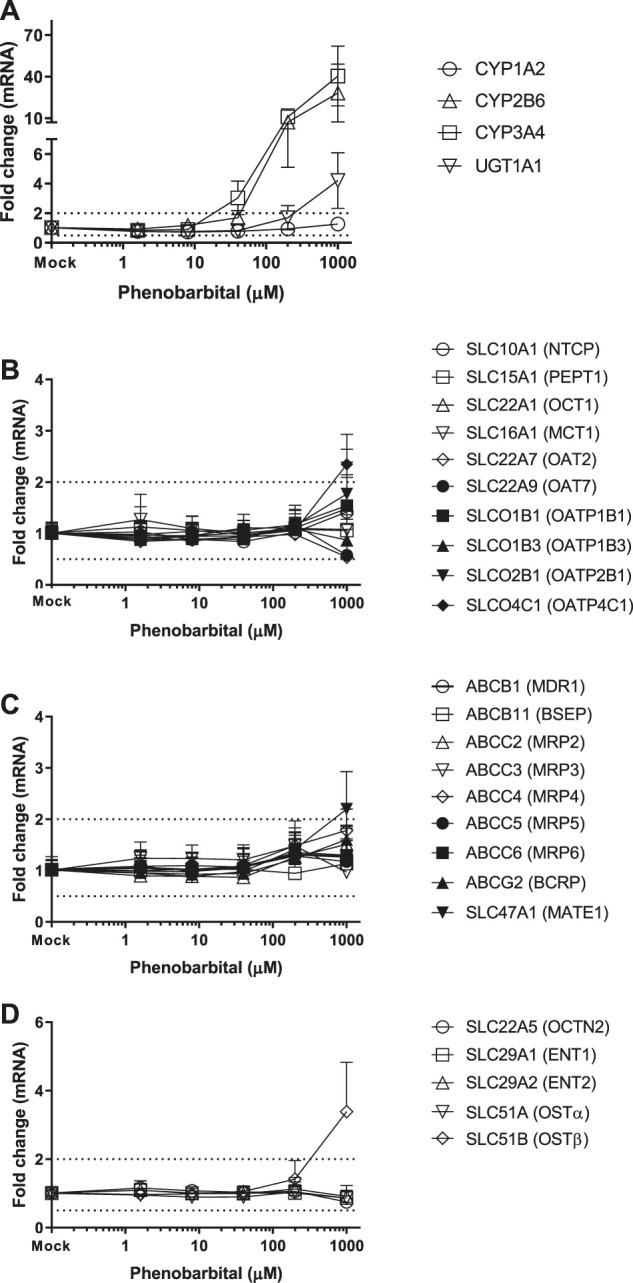
Induction of metabolizing enzyme and transporter mRNA by PB. The concentration-dependent induction studies were conducted in SCHH (Lot AOS, OQA and YTW) treated with PB at the concentrations ranging from 1.6 to 1,000 μM. The gene expressions were quantitated by qRT-PCR. Gene expression for each condition is expressed as a fold-change of mean ± standard deviation (SD) from three independent donors (each performed in triplicate). Dot lines indicate the change of 2-fold.

Twenty-four hepatic drug transporters were tested for potential gene induction in SCHH treated with prototypical activators of AhR (TCDD and OP) or CAR (CITCO and PB) for 3 days. All 24 transporter genes were found to be expressed in hepatocytes from five donors. In the US FDA guidance for industry on *in vitro* DDI studies, CYP induction is presumed when ≥ 2-fold concentration-dependent increase in CYP mRNA is observed (USFDA-Guidance-2020, 2020). Accordingly, the average fold changes of transporter genes by CAR or AhR activators in SCHH were generally less than 2-fold, with the exception of SLC51B, SLC47A1, SLCO4C1, ABCG2 and SLC10A1 genes (Table 3 and Figures 1–4). A modest (∼2-fold) and concentration-dependent induction of ABCG2 mRNA by TCDD (50 nM) was detected (Figure 1C). In contrast, marginal suppression of SLC10A1 mRNA by TCDD and OP was observed at the highest concentration treated and the gene suppression appeared to be concentration-dependent (Figure 1B and Figure 2B). A dose-dependent induction of SLC51B gene by PB and OP was detected (Figure 2D and Figure 3D), and at the highest concentration tested, the induction of SLC51B gene was about 3.4 and 6.5-fold, respectively. Additionally, about 2-fold increases of SLC47A1 and SLCO4C1 mRNA expression were also found in SCHH treated with PB at 1,000 μM. The fold induction by CITCO for all transporter genes in SCHH was generally less than 2-fold (Table 3).

## Discussion

Drugs or xenobiotics can induce the expression of metabolizing enzyme and/or transporter proteins through activating gene transcription receptors. As a result, fluctuation of systemic exposure can occur for drugs that are transported or metabolized by the induced enzymes or transporters. Thus, assessing the potentials for CYP and transporter inductions early become important de-risk steps for planning appropriate clinical DDI studies in drug research and development cycle. Primary cultures of hepatocytes are commonly used as tools to investigate induction potential of metabolizing enzyme genes, and the model is adapted to investigate transporter gene regulation (Nishimura et al., 2002; Williamson et al., 2013). However, when hepatocytes are grown in a conventional plated monolayer format, many drug metabolism and transport genes are substantially downregulated (Sun et al., 2019), which can result in false positive or negative outcomes, particularly for drug transporters that require the unique polarized architecture present in the liver. In fact, Courtois et al. (Courtois et al., 2002) observed remarkable *in vitro* induction of multidrug resistance-associated protein 2 (MRP2) mRNA and protein levels in conventional plated rat and human hepatocytes exposed to phenobarbital. Surprisingly, MRP2 induction observed *in vitro* was not reproduced in rats *in vivo* treated by the bartiturates (Courtois et al., 2002), which suggests that the positive *in vitro* induction of MRP2 could be a false positive event. Additionally, inconsistent results are reported in the literature when using the conventional plated hepatocytes for characterizing transporter gene inductions by PXR or CAR ligands, as compared with the results obtained from sandwich cultured hepatocytes (Jigorel et al., 2006). With that in mind, in the current investigation, transporter induction potential by AhR or CAR activators was accessed in SCHH to overcome the disadvantage of the conventional hepatocyte cultures suffering from loss of polarized nature of hepatocytes.

OP is a proton pump inhibitor used for the treatment of acid-related disorders. TCDD, also known as dioxin, was used as an herbicide infamously known as Agent Orange and is found in animals and humans through environmental polluted food chain. OP and TCDD are the AhR ligands (Quattrochi and Tukey, 1993). Ligands bind to the AhR receptor and form a heterodimeric complex with the AhR nuclear translocator protein, known as a transcription factor, to stimulate the transcription of CYP1A family genes. As a result, induction of CYP1A1 and UGT1A1 genes in SCHH by TCDD and OP was observed following a concentration-dependent manner (Figure 1, 2). In addition, OP, but not TCDD, also induced CYP3A4 gene to a level that was comparable to the induction of CYP1A1 (61-fold at 50 μM) (Table 3). The results agreed with the literature reports (Yoshinari et al., 2008; Donato et al., 2010), and confirmed the reliability and robustness of SCHH for investigating gene induction. Regarding the drug transporter regulation, it is known that efflux transporters ABCG2 (BCRP) and ABCB1 (P-gp) are induced by TCDD mediated AhR activation in Caco-2 and other human carcinoma cells including HepG2, LS180, LS174T and MCF7 cells (Ebert et al., 2005; Tan et al., 2010). The induction of ABCG2 mRNA in primary hepatocytes is also observed; however, the magnitude of induction is relatively small in human hepatocytes compared with those of the human carcinoma cell lines, which suggests a cell-dependent induction (Tan et al., 2010). Accordingly, a modest concentration-dependent induction of ABCG2, but not ABCB1 mRNA, by TCDD and OP was observed in SCHH (Figure 1, 2). Additionally, the SLC10A1 gene that codes the sodium-dependent taurocholate transporter protein (NTCP) for hepatic uptake of bile acid, was reduced about 2-fold by TCDD and OP (Figure 1, 2). Although we can not rule out if the maginal effects observed is due to slight cell stress at the high concentrations, this observation was supported by previous studies that TCDD can alter genes involved in cholesterol metabolism and bile acids homeostasis (Fletcher et al., 2005). Interestingly, SLC51B gene was found to be the most highly induced transporter gene by OP, but not TCDD (Figure 1, 2). SLC51A and SLC51B genes code OSTα and β protein respectively, to form the heteromeric transport protein OSTα/β. This is an important transporter in bile acid homeostasis, and can undergo adaptive regulation in the disease progression of obstructive cholestasis or primary biliary cholangitis (Ballatori et al., 2009). The OSTα/β expression can be regulated by other transcription factors including small heterodimer partner (Shp), liver receptor homolog-1 and LXR/the retinoid X receptor (RXR) heterodimer (Ballatori et al., 2009). In addition, the farnesoid X receptor (FXR), also called the bile acid receptor, and LXR may exert a coordinated role in maintaining bile acid homeostasis in the hepatocytes, which result in a novel negative feedback loop of bile acids to induce or suppress bile acid transporter genes including OSTα/β. In fact, we previously reported that SLC51B gene was also induced in hepatocytes treated with rifampin (Niu et al., 2019). Rifampicin induces CYP3A4 and CYP2C9 through activation of PXR (Maglich et al., 2002). The observation of CYP3A4 and SLC51B induction was consistent with the current literature (Novotna and Dvorak, 2014; Benson et al., 2016; Niu et al., 2019), suggesting that the induction is involved in PXR. Since AhR and PXR can share common regulation pathways (Maglich et al., 2002; Lim and Huang, 2008), our results implicate the crosstalk of AhR-PXR in human hepatocytes (Gerbal-Chaloin et al., 2006).

CITCO and PB are known as CAR direct and indirect activators, respectively. As one of the xenosensors, CAR regulates hepatic drug metabolizing enzyme genes and plays a role in mediating various hepatic functions including fatty acid oxidation, insulin signaling and biotransformation of bile acids (Gao et al., 2009; Lynch et al., 2013). For example, induction of CYP2B6 gene expression is mediated by CAR via responsive elements located in the promoter regions of CYP2B6 gene. PB does not directly bind to CAR and is considered as an indirect activator to translocate CAR into the nucleus, where it leads to transcriptional activation of target genes (Kawamoto et al., 1999; Wang et al., 2004). PB is also a human PXR ligands to induce gene expression of numerous hepatic metabolizing enzymes and transporters (Li et al., 2019). In contrast, CITCO binds to the CAR and is a direct activator of CAR. It also activates human PXR (Li et al., 2019). Here we observed that both CYP2B6 and CYP3A4 were greatly induced by PB and CITCO, and to a less extent, UGT1A1 was also induced in a concentration-dependent manner (Figure 3, 4), which is consistent with the literature reports (Li et al., 2019). Burk et al. reported that CAR can also regulate MDR1 gene in cells stably expressing CAR (Burk et al., 2005). Our findings suggested that CITCO and PB may also activate the PXR receptor to induce CYP3A gene (Novotna and Dvorak, 2014; Li et al., 2019). Controversially, neither CITCO nor PB induced the ABCB1 (MDR1) gene in SCHH (Figure 3 and 4). This suggests that transporter induction can be cell line (or organ) specific. Interestingly, SLC51B gene was also induced by PB, but not CITCO (Table 3). Together with the SLC51B induction by OP, PB and rifampin that are PXR ligands (Benson et al., 2016; Niu et al., 2019), the SLC51B induction appears to likely not be mediated by CAR/AhR receptors. In addition, OSTβ heterodimerizes with OSTα to form the functional transporter complex, OSTα/β. Therefore, the outcome of SLC51B (OSTβ) induction, but not SLC51A (OSTα), remains unclear and further investigation on OSTβ induction is warranted.

Collectively, according to the regulatory guidance that a concentration-dependent 2-fold increase in mRNA is considered the threshold for a positive *in vitro* induction signal (USFDA-Guidance-2020, 2020), our results revealed that except SLC51B gene, hepatic transporter gene regulations by CAR and AhR ligands were generally marginal, and significantly lower than the inductions of metabolizing enzyme genes. The current findings suggest that the assessment of transporter gene inductions is not required when a drug does not remarkably induce metabolizing enzyme genes by CAR and AhR activation.

## Data Availability Statement

The datasets presented in this study can be found in online repositories. The names of the repository/repositories and accession number(s) can be found in the article/Supplementary Material.

## Author Contributions

YL, CN, and BS participated in research design. CN conducted experiments. YL and CN performed data analysis. YL, CN, and BS wrote or contributed to the writing of the manuscript.

## Conflict of Interest

All authors were employed by Gilead Sciences Inc.

## Abbreviations

CAR, the constitutive androstane receptor; AhR, the aryl hydrocarbon receptor. qRT-PCR, real-time quantitative reverse transcription polymerase chain reaction. SLC transporters, solute carrier transporters; ABC transporters, ATP-binding cassette transporters. TCDD, 2,3,7,8-tetrachlorodibenzo-p-dioxin; OP, omeprazole; CITCO, 6-(4-chlorophenyl)imidazo[2,1-b][1,3]thiazole-5-carbaldehyde O-(3,4-dichlorobenzyl)oxime; PB, phenobarbital.
