# Supplementary material for: Transporter Gene Regulation in Sandwich Cultured Human Hepatocytes Through the Activation of Constitutive Androstane Receptor (CAR) or Aryl Hydrocarbon Receptor (AhR)
Source: Front Pharmacol. 2021 Jan 21;11:620197. doi: 10.3389/fphar.2020.620197 (PMC7859440; doi:10.3389/fphar.2020.620197)
Supplement: Supplementary file 1 [file datasheet1.pdf]

Supplementary material

Transporter gene regulation in sandwich cultured human hepatocytes through the activation of constitutive androstane receptor (CAR) or aryl hydrocarbon receptor (AhR)

Congrong Niu, Bill Smith and Yurong Lai[#]

Drug Metabolism, Gilead Sciences Inc., Foster City, CA 94404

Running title: Transporter gene regulations by AhR and CAR ligands

[#]Corresponding author and contact information:

Mailing address: Gilead Sciences Inc. 333 Lakeside Dr. Foster City, CA 94404
Phone: (650) 522-1629. E-mail: yurong.lai@gilead.com

Figure 1. The lot differences of gene induction by CITCO (5 µM), PB (1000 µM), OP (50 µM) and TCDD (50 nM) in SCHH. The gene expressions were quantitated by real-time quantitative reverse transcription polymerase chain**.** Gene expression for each condition is expressed as a fold-change of mean ± standard deviation (SD) from three independent donors (each performed in triplicate). Dot lines indicate the change of 2-fold.

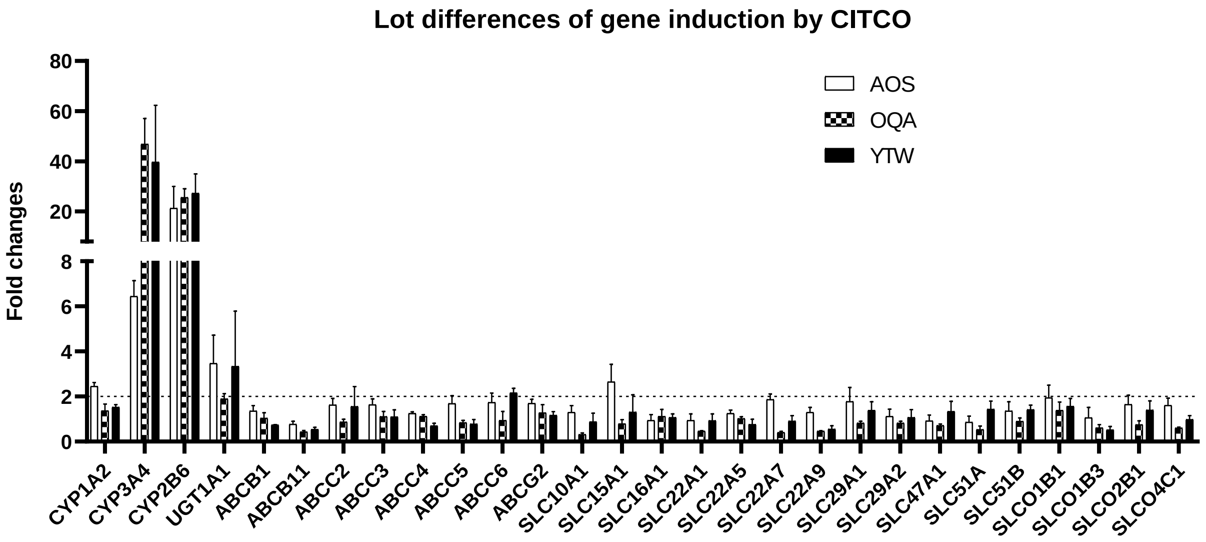

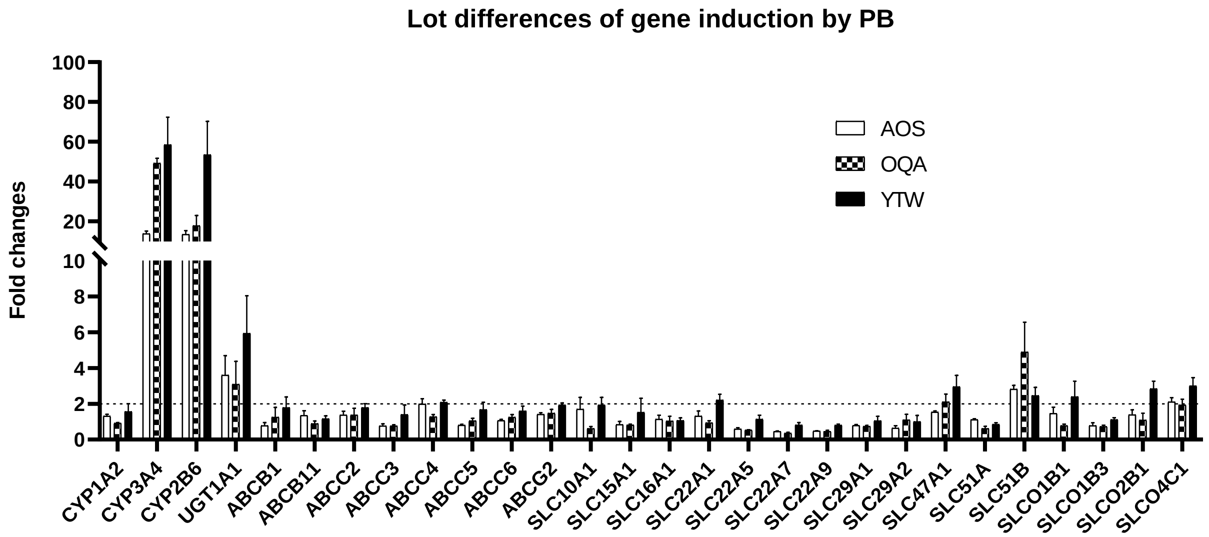
Lot differences of gene induction by PB
Fold changes
AOS
OQA
YTW
CYP1A2
CYP3A4
CYP2B6
UGT1A1
ABCB1
ABCB11
ABCC2
ABCC3
ABCC4
ABCC5
ABCC6
ABCG2
SLC10A1
SLC15A1
SLC16A1
SLC22A1
SLC22A5
SLC22A7
SLC22A9
SLC29A1
SLC29A2
SLC47A1
SLC51A
SLC51B
SLCO1B1
SLCO1B3
SLCO2B1
SLCO4C1

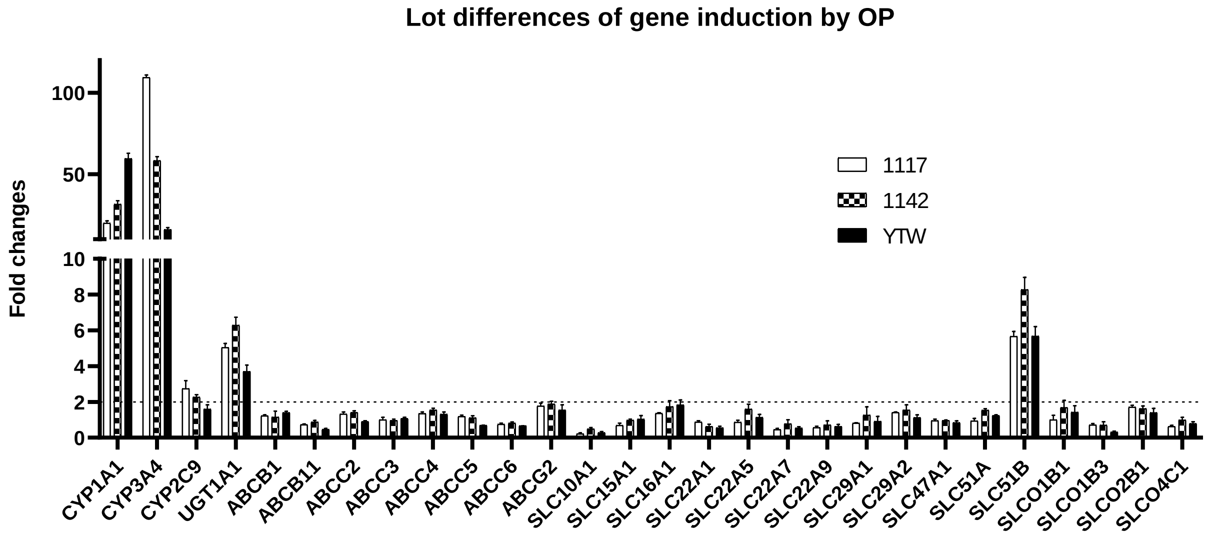
Lot differences of gene induction by OP
Fold changes
1117
1142
YTW
CYP1A1
CYP3A4
CYP2C9
UGT1A1
ABCB1
ABCB11
ABCC2
ABCC3
ABCC4
ABCC5
ABCC6
ABCG2
SLC10A1
SLC15A1
SLC16A1
SLC22A1
SLC22A5
SLC22A7
SLC22A9
SLC29A1
SLC29A2
SLC47A1
SLC51A
SLC51B
SLCO1B1
SLCO1B3
SLCO2B1
SLCO4C1

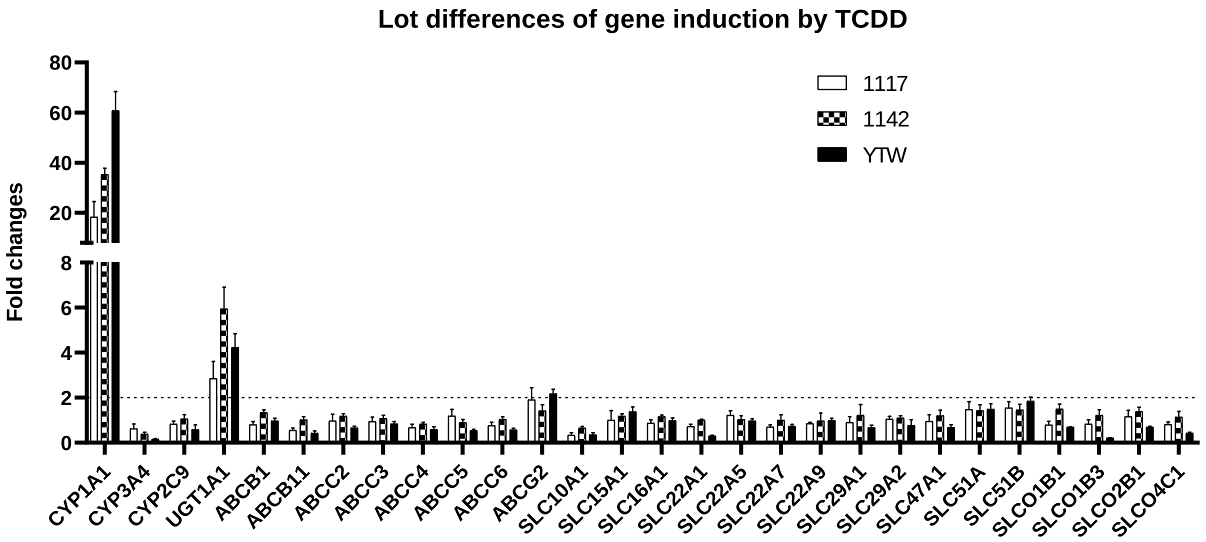

Table 1. The Ct values of β-actin and GAPDH in sandwich cultured hepatocytes treated with inducers (lot YTW)

TCDD (nM)	β-actin			GAPDH			Omeprazole (μM)	β-actin			GAPDH		
0	20.46	20.02	20.25	21.06	20.84	20.26	0	20.54	20.94	20.83	20.51	20.56	20.86
0.08	20.86	20.43	20.69	21.44	21.98	21.62	0.08	21.74	21.98	21.87	21.38	21.98	21.45
0.4	19.60	19.57	19.57	20.06	20.26	19.47	0.4	19.42	19.84	20.07	20.48	20.81	20.67
2	20.22	20.11	20.17	21.60	21.28	21.20	2	20.12	20.70	20.76	21.32	21.28	21.81
10	19.72	19.93	19.98	21.19	21.17	20.94	10	20.00	20.27	20.68	21.29	21.03	21.64
50	19.31	19.38	19.67	19.86	20.38	19.80	50	19.25	19.49	19.45	20.20	20.16	19.87
CITCO (μM)	β-actin			GAPDH			phenobarbital (μM)	β-actin			GAPDH		
0	20.22	19.57	19.94	20.21	20.45	20.34	0	19.99	19.46	19.72	19.86	19.75	19.92
0.008	19.90	19.44	19.90	20.59	19.17	19.43	1.6	19.93	19.32	19.46	19.64	19.14	19.77
0.04	20.87	20.46	19.71	20.54	19.75	19.91	8	19.13	19.13	19.08	19.90	19.43	19.55
0.2	18.98	19.02	19.26	20.34	19.68	20.06	40	19.03	19.32	19.16	19.30	19.31	19.25
1	20.66	20.52	20.12	21.04	19.98	20.21	200	19.37	19.22	19.18	19.47	19.66	19.01
5	19.92	20.86	20.29	20.36	20.73	19.93	1000	20.13	19.93	19.86	20.13	20.11	19.50
